# Influence of job demands on implicit absenteeism in Chinese nurses: mediating effects of work–family conflict and job embeddedness

**DOI:** 10.3389/fpsyg.2023.1265710

**Published:** 2023-10-23

**Authors:** Yujie Zhang, Shanyan Lei, Liying Chen, Fang Yang

**Affiliations:** ^1^School of Humanities and Management, Zhejiang Chinese Medical University, Hangzhou, China; ^2^Second School of Clinical Medicine, Zhejiang Chinese Medical University, Hangzhou, China; ^3^Department of Nursing, Weifang People’s Hospital, Weifang, China

**Keywords:** implicit absenteeism, nurses, productivity loss, job embeddedness, China

## Abstract

**Purpose:**

It has been widely noted that implicit absenteeism is common among nurses, with job demand influencing it. Theoretically, work–family conflict and job embeddedness may help link job demands to implicit absenteeism. However, the mediating effects of the two on the association between job demands and implicit absenteeism remain unclear. Thus, this study aims to explore the association between nurses’ job demands and implicit absenteeism, and the chain mediating effect of work–family conflict and job embeddedness in this relationship.

**Patients and methods:**

Data were collected from 1,420 nurses from five tertiary public hospitals in China. They were asked to respond to a questionnaire asking about job demands, implicit absenteeism, work–family conflict, and job embeddedness. The data were coded and analyzed using IBM SPSS version 21.0. Descriptive analysis, *t*-test, one-way ANOVA, hierarchical multiple regression analysis, and bootstrapping were used to analyze the extracted data.

**Results:**

The mean score for implicit absenteeism was 17.75 ± 5.60. There was a significant correlation (*p* < 0.05) between nurses’ job demands, work–family conflict, job embeddedness, and implicit absenteeism. Nurses’ job demands directly influenced implicit absenteeism and indirectly influenced implicit absenteeism through the mediating effects of work–family conflict and job embeddedness. Furthermore, work–family conflict and job embeddedness have a chain effect on the association between job demands and implicit absenteeism.

**Conclusion:**

The study found that nurses’ job demands directly and positively influence implicit absenteeism, and indirectly influence implicit absenteeism through single and chain mediating effects of work–family conflict and job embeddedness.

## 1. Introduction

Implicit absenteeism (IA) refers to the phenomenon in which individuals work less efficiently because of physiological, psychological, and social factors. Although they are present at work, their work engagement is reduced, or their work motivation is lower (Aronsson et al., 2000; Burmeister et al., 2019). Previous research suggested that productivity loss attributed to IA is much higher than that due to absence from sickness (Liu et al., 2019; Sun et al., 2019). Implicit absenteeism is more common among healthcare workers with a significantly higher incidence than in other occupational types (Aysun and Bayram, 2017; Jin et al., 2022). Among Chinese nurses, heavy workloads, irregular working hours, and fierce competition for title promotion have led to IA being more prevalent among clinical nurses. China’s National Mental Health Report states that the incidence of IA among nurses is 3–4 times higher than that of the average corporate employee (Fu and Zhang, 2019).

Several studies have indicated that nurses’ IA is connected with patient safety, care quality, and work efficiency (Letvak et al., 2012; Labrague et al., 2020; Déry et al., 2022; Zeighami et al., 2023). It can increase the incidence of negative events such as patient falls and infections. Prolonged IA may also lead to deterioration of the nurses’ health, which may affect their productivity or personal performance, resulting in greater financial losses to hospitals and patients. Therefore, it is important to investigate the inner mechanisms of IA among nurses and to take effective interventions to reduce its occurrence.

Job demands (JD) are defined as occupational skills that involve consistent physical and mental effort (Bakker et al., 2004). According to the personal-environmental matching theory and the ability-pressure model, the matching of an individual’s abilities, values, expectations, or goals with the environment can produce different outcomes and effects (Cable and Derue, 2002; Nahemow et al., 2016). When personal traits do not match the characteristics of the environment in which they are located, it can cause negative consequences like stress to the individual. Simultaneously, according to the JD-R model, there is indeed a “depletion” path for the impact of work on individuals (Bakker et al., 2004). The work places physical, social or organizational demands on the individual. Examples include work overload, time pressure, interpersonal demands, and demands on emotional performance. In order to comply with these demands, individuals have to make constant physical or psychological efforts. Thus, these high demands have a negative effect on the psychology or physiology of individuals. Ultimately, they have a resultant negative impact.

Nurses face a heavy workload and fierce competition for promotions. As medical service workers, they also have to be strongly conscious of service, dedication, among other aspects of their jobs. Negative behaviors like IA will inevitably occur when nurses find that their job competencies do not match their JDs and that the work environment does not match their personal needs or preferences. Demerouti et al. confirmed an association between JD and nurses’ IA (Demerouti et al., 2009). It has been noted that JD has a positive effect on nurse IA. However, the mechanism of action remains to be studied. Therefore, we propose the first hypothesis:

*Hypothesis 1*: JD will significantly predict IA (JD → IA).

Work–family conflict (WFC) is the uncontrollable, incompatible, and irreconcilable negative experience of various characters in individuals’ work and family domains and is divided into work-to-family conflict and family-to-work conflict (AlAzzam et al., 2017). Ghislieri et al. and Rhéaume found an association between JD and WFC: the higher the JD, the higher the level of WFC (Ghislieri et al., 2017; Rhéaume, 2022). Some studies have shown that failure to respond effectively to JD can create an imbalance between work and family, resulting in a series of possible adverse consequences (Frone, 2000; Amstad et al., 2011). Previous research has shown that WFC among nurses is associated with a variety of negative outcomes. Prior studies have found that nurses’ WFC had an impact on work-related variables such as task performance, job satisfaction, and turnover intentions (Cortese et al., 2010; Wang and Tsai, 2014; Yildiz et al., 2021; Moreira et al., 2023). Furthermore, work–family conflict was associated with physical and mental health-related variables like depression and neck and back pain among nurses (Baur et al., 2018; Cheng et al., 2019; Lee et al., 2022). However, few studies have demonstrated the correlation between WFC and IA, and it remains to be seen whether WFC mediates the association between JD and IA. Therefore, we propose the second hypothesis:

*Hypothesis 2*: JD will influence IA through the mediating effect of WFC (JD → WFC → IA).

Job embeddedness (JE) is defined as the degree of closeness between an employee and the organization and the difficulty for the employee in leaving the organization (Mitchell et al., 2001). It can provide a valuable research perspective for explaining the mechanism of action between JD and IA. When nurses’ JD is low, they may match their job competencies with their job tasks better. This results in higher satisfaction with the organization and a higher level of closeness between the organization and colleagues, i.e., higher JE. Furthermore, the outcome variables of JE focus on attitudes or behavior-related variables like turnover intention, work performance, and organizational citizenship behavior (Afsar et al., 2018; Kapil and Rastogi, 2018; Lee and Huang, 2019). However, the association between JE and IA remains unclear, and whether JE mediates this relationship has not been investigated. Therefore, we propose the third hypothesis:

*Hypothesis 3*: JD will influence IA through the mediating effect of JE (JD → JE → IA).

Based on the hypothesis of limited allocation of individual resources, work and family, as important domains of individual life, may compete with each other for individual’s limited resources, and the competition between them may trigger an individual’s psychological conflict (Grawitch et al., 2010). Based on the theory of work-family boundary management, WFC may lead to the confusion of the boundary between an individual’s work and family domains, which in turn may have negative effects (Clark, 2000). Studies have shown that individuals encounter WFC or contradictions, and whether the problem can be resolved directly affects the employees’ work performance and turnover intention. According to the work-family border theory, the degree of JE of nurses is influenced by WFC. A prior study found that WFC has a negative impact on JE - the higher the degree of WFC, the lower the degree of JE (Ng and Feldman, 2014). Therefore, we propose the fourth hypothesis of this study:

*Hypothesis 4*: WFC and JE will jointly play an intermediary role in the relationship between JD and IA (JD → WFC → JE → IA).

**Figure 1:**
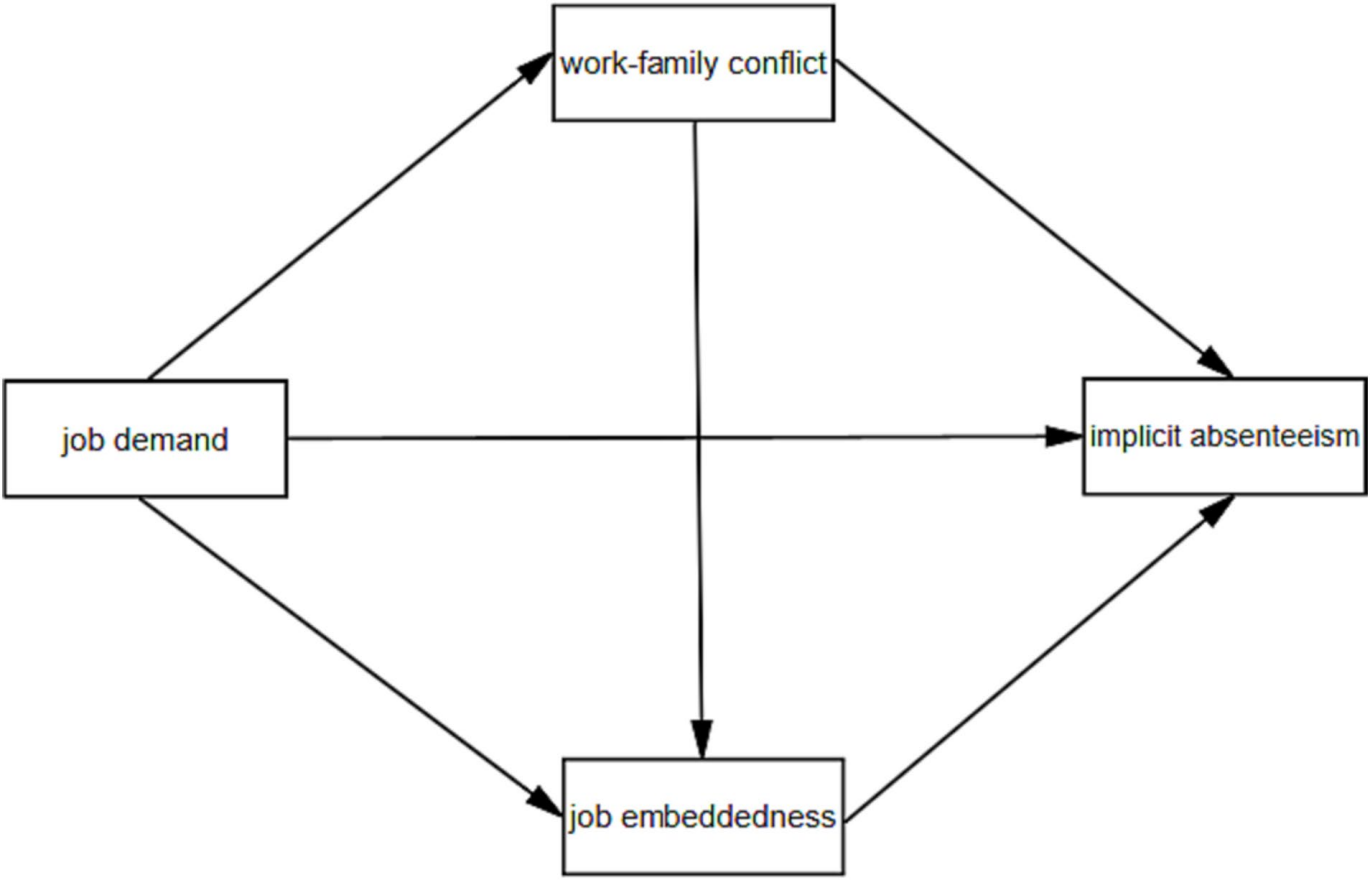
Hypothesis model.

Based on theories and the existing literature, taking nurses in tertiary public hospitals as the study participants, this study performed a hypothesis model (Figure 1) to investigate the mechanisms of WFC and JE between JD and IA to provide theoretical references for the adoption of targeted measures.

## 2. Materials and methods

### 2.1. Study design

Cross-sectional research was conducted to report the chain-mediating effect of WFC and JE between nurses’ JD and IA.

### 2.2. Participants and data collection

A two-stage sampling method was adopted in the period between October and December 2021. In the first stage, five tertiary public hospitals in Weifang City, Shandong Province, were selected as sampling units using the grasping random ball method. In the second stage, several departments were randomly selected in each hospital according to the principle of 30% equal proportion, and nurses on duty in the sampled departments on that day participated in the survey. The inclusion criteria were as follows: (i) active registered nurses; (ii) more than 1 year of experience in clinical nursing; and (iii) informed consent and voluntary participation in this study. The exclusion criteria were as follows: (i) enrolled nurses, nurse interns, advanced practice nurses, and (ii) nurses who were not on duty during the survey period. Of the 1,542 nurses, 1,420 completed the questionnaire effectively. The response rate was 0.921. The participants were assured of the anonymity of their responses, and that their refusal to participate did not have any negative consequences.

### 2.3. Instruments

#### 2.3.1. Demographic questionnaire

Demographic characteristics included gender, age, educational level, marital status, type of employment, monthly income, health status, and work intensity.

#### 2.3.2. Job demands scale

This scale, developed by Li et al. (2014), includes six dimensions: workload; job-sharing; and emotional, environmental, psychological, and timing demands, with a total of 19 items. All items were described negatively, and the questionnaire was scored on a 5-point Likert scale. Higher scores indicate higher JD.

#### 2.3.3. Work–family conflict scale

This scale was developed by Grzywacz and Marks (2000) and translated by Zeng and Yan (2013). It comprises two dimensions: work-to-family conflict and family-to-work conflict, with a total of eight items. The scoring was based on a 5-point Likert scale, with a self-assessment scale ranging from “1” to “5,” indicating “*strongly disagree*,” “*relatively disagree*,” “*uncertain*,” “*agree*,” and “*strongly agree*,” respectively. The higher the score, the stronger the degree of WFC.

#### 2.3.4. Global job embeddedness scale

This unidimensional scale with seven entries was developed by Crossley et al. (2007) and is widely used in China. It is rated on a 5-point Likert scale, with higher scores indicating higher levels of JE.

#### 2.3.5. Stanford presenteeism scale

Developed by the Stanford University (Koopman et al., 2002), United States, and translated and revised by Zhao et al. (2010), this scale contains six entries and uses a 5-point Likert scale, where entries 5 and 6 are reverse-scored. The higher the score, the higher the level of IA. The Chinese version of the scale was proven to have good reliability, with Cronbach’s alpha coefficients for each entry ranging between 0.76 and 0.90.

### 2.4. Data analysis

The SPSS (version 22.0) and AMOS software were used for data input and statistical analyzes. The reliability of scales were tested in terms of internal consistency reliability and composite reliability. Internal consistency reliability was measured by Cronbach’s alpha coefficients. The scale’s validity was tested in terms of structural validity, convergent validity, and discriminant validity. Structural validity was tested using confirmatory factor analysis. Convergent validity and discriminant validity were measured on the basis of average variance extracted and factor loading. The measurement data were expressed as mean ± standard deviation, and the count data were described by frequency and percentage (%). All data were checked for normality using QQ plots and histograms and were found to be approximately normally distributed. One-way ANOVA was performed using an independent *t*-test and one-way ANOVA. Pearson’s correlation analysis was used to explore the correlations among JD, WFC, JE, and IA. Hierarchical multiple regression analysis and bootstrapping were used to test the mediating effects. The significance level was set at *p* < 0.05.

## 3. Results

### 3.1. Demographic characteristics of participants

Table 1 shows the sociodemographic and occupational characteristics of the participants. The majority of the participants were women (95.6%), aged between 31 and 40 years (44.4%). They had a bachelor’s degree or higher (89.2%), were informally employed (83.2%), and received an average monthly income between RMB 5001–7,000 (35.6%). Less than half of the nurses (38.7%) had an average health status, and more than half of the nurses (52.0%) considered their work to be more intensive.

**Table 1:** Differences in variables based on demographic characteristics.

Characteristic	*N* (%)	Mean ± SD	*t/F*	*p*
Sex				
Male	63 (4.4)	16.86 ± 5.90	−1.29	0.20
Female	1,357 (95.6)	17.79 ± 5.58		
Age(year)				
≤30	548 (38.6)	17.36 ± 5.41	2.84	0.04
31–40	630 (44.4)	18.23 ± 5.63		
41–50	195 (13.7)	17.32 ± 5.86		
≥51	47 (3.3)	17.55 ± 5.83		
Educational level				
Junior college and below	153 (10.8)	16.54 ± 5.21	4.75	0.009
Undergraduate	1,251 (88.1)	17.91 ± 5.62		
Postgraduate or above	16 (1.1)	16.19 ± 5.50		
Marital status				
With spouse/partner	336 (88.2)	17.61 ± 5.47	−0.50	0.62
Without spouse/partner	1,084 (11.8)	17.79 ± 5.64		
Monthly income (RMB)				
<3,000	89 (6.3)	17.57 ± 4.66	3.16	0.01
3,000 ~ 5,000	269 (18.9)	16.94 ± 5.14		
5,001 ~ 7,000	505 (35.6)	17.55 ± 5.66		
7,001 ~ 9,000	363 (25.6)	18.31 ± 5.91		
>9,000	194 (13.7)	18.39 ± 5.69		
Healthy status				
Very unhealthy	35 (2.5)	19.77 ± 6.93	19.03	<0.001
Less healthy	333 (23.5)	19.26 ± 5.40		
General healthy	550 (38.7)	18.16 ± 5.20		
More healthy	430 (30.3)	16.19 ± 5.66		
Very healthy	72 (5.1)	15.96 ± 5.66		
Work intensity				
Very low/rarely	34 (2.4)	14.29 ± 6.41	23.93	<0.001
Commonly	419 (29.5)	16.40 ± 5.01		
Higher	739 (52.0)	18.06 ± 5.44		
Very high	228 (16.1)	19.74 ± 6.15		

### 3.2. Assessment of the measurement instruments reliability and validity

The Cronbach’s alpha coefficients for the variables ranged between 0.738 and 0.930, and the CR values ranged between 0.739 and 0.931. Both exceeded the recommended critical value of 0.7, indicating good reliability of the scales (Table 2). The results of the confirmatory factor analysis indicated that JDs, WFC, JE, and IA had a six-factor, two-factor, one-factor, and one-factor structure, respectively. The model fit was good (Hu and Bentler, 1999), and the factor loadings were all greater than 0.5 (Supplementary material). The scales had a good construct validity.

**Table 2:** Assessment of the measurement instruments reliability and validity.

Variables	1	2	3	4	5	6	*AVE*	Cronbach’s α	*CR*
*Job demands*									
1 Workload demands	–						0.532	0.843	0.849
2 Psychological demands	0.319	–					0.587	0.738	0.739
3 Emotional demands	0.156	0.516	–				0.654	0.841	0.848
4 Environmental demands	0.605	0.267	0.146	–			0.579	0.802	0.804
5 Job-sharing demands	0.593	0.287	0.188	0.489	–		0.787	0.916	0.917
6 Timing demands	0.688	0.314	0.146	0.581	0.640	–	0.509	0.755	0.757
*AVE* square root	0.729	0.766	0.809	0.761	0.887	0.713			
*Work–family conflict*									
1 Work-to-family conflict	–						0.756	0.922	0.925
2 Family-to-work conflict	0.458	–					0.771	0.930	0.931
*AVE* square root	0.869	0.878							
*Job embeddedness*							0.541	0.872	0.890
*Implicit absenteeism*							0.535	0.850	0.869

The average extracted variance values for each variable were all above 0.5. The factor loading values were all above 0.5 (Table 2). This indicated that the scales had good convergent validity. The square root of the average variance extracted values of the dimensions of JDs and WFC were greater than their correlation coefficients with the other dimensions. The correlation coefficients between the dimensions were less than 0.85 (Table 2). This indicated that JDs and WFC had good discriminant validity.

### 3.3. Differences in variables based on demographic characteristics

We found differences in the level of IA among nurses of different ages, education, monthly income, work intensity, and health status (*p* < 0.05), with relatively higher levels of IA among nurses aged 31–40 years, with a bachelor’s degree, higher monthly income, very poor health status, and very high work intensity (Table 1).

### 3.4. Correlation analysis of JD, WFC, JE, and IA

The results showed that JD was positively associated with WFC and IA (*p* < 0.01); JD was negatively correlated with JE (*p* < 0.01); WFC was positively correlated with IA (*p* < 0.01); WFC was negatively associated with JE (*p* < 0.01) and JE was negatively related to IA (*p* < 0.01) (Table 3).

**Table 3:** Pearson correlation coefficient among variables.

Variables	*M*	SD	JD	WFC	JE	IA
JD	69.16	12.10	1			
WFC	22.53	6.54	0.55^**^	1		
JE	23.54	5.98	−0.28^**^	−0.40^**^	1	
IA	17.75	5.60	0.32^**^	0.38^**^	−0.26^**^	1

^**^ indicates *p* < 0.01.

### 3.5. The chain mediating effect of WFC and JE between JD and IA

**Figure 2:**
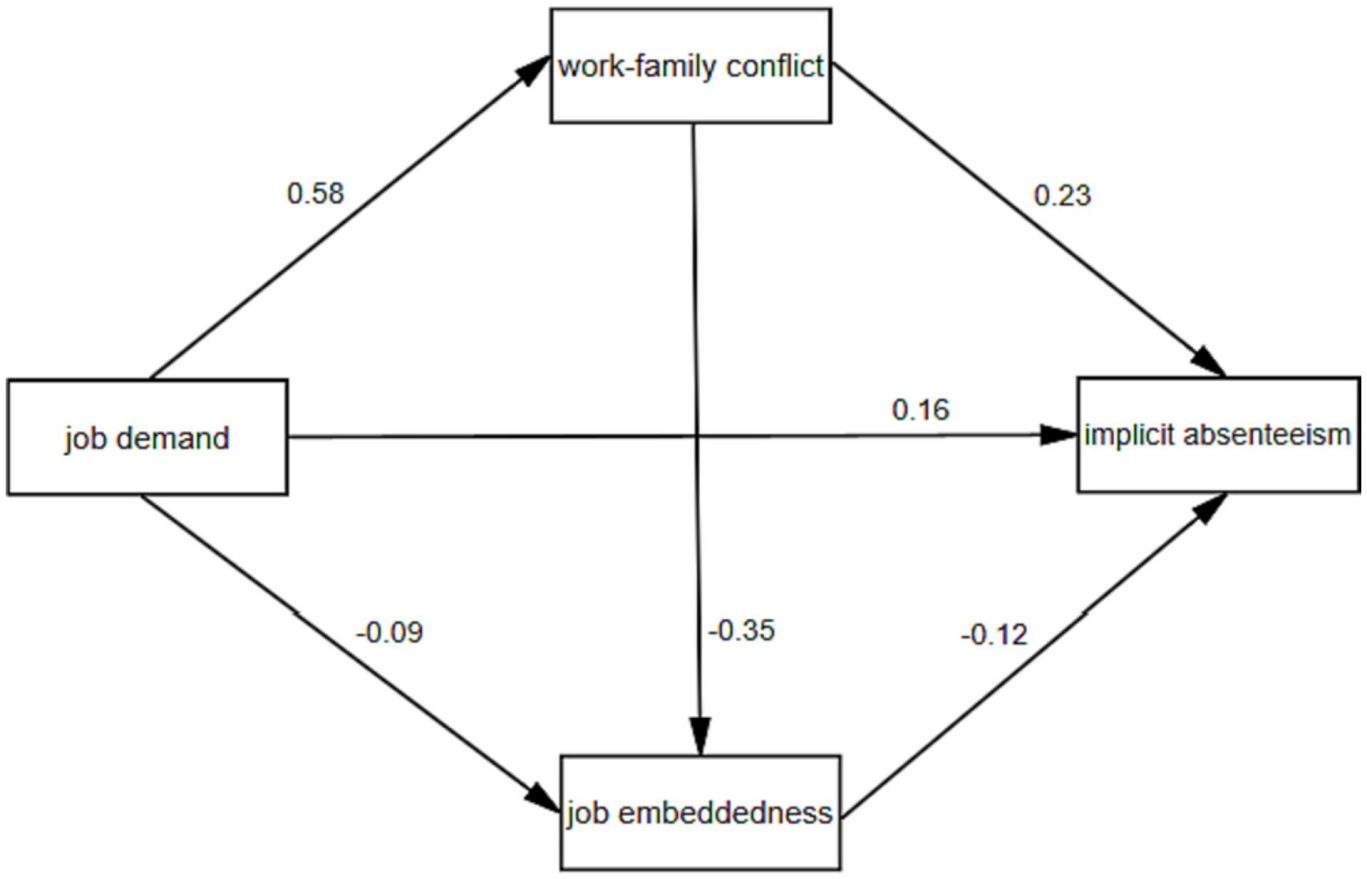
Multiple mediating models of WFC and JE between JD and IA.

Model 6 in the PROCESS macro program developed by Preacher and Hayes (2004) was used to test the chain-mediating effect of WFC and JE between JD and IA. Age, education level, monthly income, work intensity, and health status were used as control variables. The results showed that JD positively predicted IA (*β* = 0.27, *p* < 0.001), positively influenced WFC (*β* = 0.49, *p* < 0.001), and negatively predicted JE (*β* = −0.12, *p* < 0.001); while WFC negatively predicted JE (*β* = −0.33, *p* < 0.001). When JD, WFC, and JE were entered into the regression equation simultaneously, JD (*β* = 0.13, *p* < 0.001), WFC (*β* = 0.23, *p* < 0.001), and JE (*β* = −0.12, *p* < 0.001) predicted IA (Table 4 and Figure 2).

**Table 4:** Results of hierarchical regression analysis.

Model	Variables		Fit indices		Coefficient significance		
	Dependent variable	Independent variable	*R^2^*	*F*	*β*	*SE*	*t*
Model 1	IA	JD	0.12	31.77^***^	0.27	0.01	8.41^***^
Model 2	WFC	JD	0.37	139.99^***^	0.49	0.01	17.84^***^
Model 3	JE	JD	0.18	43.45^***^	−0.12	0.02	−3.34^***^
		WFC			−0.33	0.03	−10.79^***^
Model 4	IA	JD	0.18	38.04^***^	0.13	0.02	3.62^***^
		WFC			0.23	0.03	7.26^***^
		JE			−0.12	0.02	−4.63^***^

^***^ indicates *p* < 0.001.

Further results showed that indirect path 1 (JD → WFC → IA) had an indirect effect value of 0.112; indirect path 2 (JD → JE → IA) had an indirect effect value of 0.014; indirect path 3 (JD → WFC → JE → IA) had an indirect effect value of 0.020. The upper and lower limits of Bootstrap 95% confidence intervals for the above paths do not contain 0, indicating that all above paths are significant, with effect values of 41.18, 5.15, and 7.35% of the total effect, respectively (Table 5).

**Table 5:** Results of bootstrapping mediation effect examination.

Path	Effect	SE	Bootstrap 95% CI		Proportion of effect
			Lower	Upper	
JD → WFC → IA	0.112	0.018	0.077	0.149	41.18%
JD → JE → IA	0.014	0.006	0.004	0.027	5.15%
JD → WFC → JE → IA	0.020	0.006	0.009	0.032	7.35%
Total indirect effects	0.146	0.019	0.110	0.184	53.68%

## 4. Discussion

The objective of this study was to explore the association between nurses’ JD and IA and to clarify whether WFC and JE have a chain-mediating effect on the association. This study also provides a new perspective on the association between nurses’ JD and IA. The results showed that the nurses’ IA score was 17.75, which was slightly higher than that reported by Jin et al. (2022) and Ren et al. (2019). The median score was used as the cutoff point to classify high and low levels of IA. The findings revealed that 54.1% of nurses had a relatively higher level of IA. This suggests that IA among nurses is relatively common in China. It suggests that preventing and intervening in the implicit absenteeism of nurses is essential.

From the perspective of JD, this study is similar to previous research findings in regard to the fact that there is an association between JD and IA (Framke et al., 2019; Aronsson et al., 2021). The tertiary public hospitals in China are large medical institutions in the region and nurses have a heavy workload (Wu et al., 2018). Moreover, competition for promotion among nurses is fierce, and there is more pressure on professional knowledge, learning, and research. Simultaneously, the nurses have to face patients with different diseases and personality types in their daily work and meet the reasonable needs of patients as much as possible. Along with the increasing awareness of patients’ self-advocacy, if they cannot maintain a high degree of concentration, there is a higher risk of nurse–patient disputes or medical errors in cases of negligence (Tucker et al., 2015). This places high psychological and emotional demands on the nurses. Nurses face heavy workloads and psychological and emotional demands, especially those with a lower level of clinical technical ability and work experience, such as new nurses and nurses with a lower education level. Their physical and psychological load is heavy, and they may experience corresponding physical or psychological problems, which affect their work efficiency, resulting in IA.

The results suggested that WFC mediated the association between nurses’ JD and IA. The high JD of nurses in tertiary public hospitals and the necessity of working as a means to make a living can cause them to focus their time and energy primarily on work. Most nurses are female. Owing to the influence of traditional Chinese ideologies, women bear heavy family responsibilities in raising children and supporting older adults (Dousin et al., 2019). Moreover, most nurses’ family members are “double workers,” hence, their spouses and family members have limited sharing of family responsibilities, which may lead to conflicts between family and work nurses. Based on the work-family boundary theory, family-work dissonance can affect an individual’s work behavior. For individuals experiencing WFC, it is difficult to make a smooth transition between the boundaries of the work and family life domains. This in turn produces negative impact outcomes (Ernst Kossek and Ozeki, 1998; Clark, 2002). It can be said, WFC may affect nurses’ physical and mental health, making it difficult for them to maintain a high level of physical and mental engagement at work, leading to impaired productivity and IA.

This study confirmed the mediating effect of JE on JD and IA among nurses. It validates the possibility of exploring the effect of JD on IA from the perspective of JE. In China, nurses in tertiary public hospitals have relatively better job remuneration, stability, and security than those in low-grade hospitals, private hospitals, or some other occupations. When the level of JD is lower, nurses may have more positive experiences at the work level, which gives them a stronger sense of occupational group identity and organizational belonging, and a higher degree of closeness with the organization and colleagues, i.e., a higher degree of JE. Nurses with a higher degree of JE are more likely to take sick leaves when they have physical or psychological problems, which can effectively influence IA (Ren et al., 2019). It is possible that when they attend work with physical discomfort, due to their sense of responsibility toward the organization, colleagues, and patients, nurses may maintain more positive emotions at work and avoid implicit absences as much as possible.

This study also found that WFC and JE had a chain-mediating effect between nurses’ JD and IA. This result was similar to that reported by Yang and Chen (2020). On the other hand, Ma et al. (2014) verified the relationship between work-family facilitation and JE from the perspective of work-family facilitation. Ma et al.’s study of corporate employees found that work-family facilitation had a positive effect on JE. This result is also in line with the work-family balance theory, which states that when nurses have difficulty balancing work and family life, the degree of JE decreases as the level of WFC increases. In particular, family-to-work conflict can make it difficult for nurses to receive understanding and support from family members so they work with a lower degree of commitment. Inevitably, it is easy to experience IA in the long run, such as being out of work or attending work without a sense of responsibility.

In this study, nurses in five tertiary public hospitals were selected as respondents, hence the comprehensiveness of the data may be somewhat limited. Therefore, it is difficult to infer a causal association between these variables in the cross-sectional study. Large-scale, multicenter prospective research is necessary to validate the study findings. Additionally, this study adopted a questionnaire survey to collect relevant data and information, which is susceptible to the influence of subjective factors of survey respondents. Because of the dynamic nature of IA, the data and information may have some limitations in reflecting the IA of nurses.

Our study constructed a chain-mediation model to explore the association among JD, WFC, JE, and IA. We found that nurses’ IA was high, and that nurses’ JD could directly and positively influence IA, while WFC and JE had a single and chained mediating role between JD and IA. The practical implications of this study are prominent, and the results can be used to develop strategies for mitigating IA among nurses, particularly among Chinese nurses. The results suggested that hospital managers should effectively alleviate physical and mental stress caused by JD on nurses by reasonably reducing their workload, promptly relieving their psychological stress, and optimizing their shift patterns. Furthermore, nurses’ families should focus on creating a good family atmosphere to enhance their understanding and support for nursing work.

## Data availability statement

The raw data supporting the conclusions of this article will be made available by the authors, without undue reservation.

## Ethics statement

The studies involving humans were approved by Ethics Committee of Weifang People’s Hospital. The studies were conducted in accordance with the local legislation and institutional requirements. The participants provided their written informed consent to participate in this study.

## Author contributions

YZ: Writing – original draft, Writing – review & editing. SL: Writing – original draft, Writing – review & editing. LC: Writing – original draft, Writing – review & editing. FY: Writing – original draft, Writing – review & editing.
