# Supplementary material for: Influence of job demands on implicit absenteeism in Chinese nurses: mediating effects of work–family conflict and job embeddedness
Source: Front Psychol. 2023 Oct 23;14:1265710. doi: 10.3389/fpsyg.2023.1265710 (PMC10627013; doi:10.3389/fpsyg.2023.1265710)
Supplement: Supplementary file 1 [file Table_1.DOC]

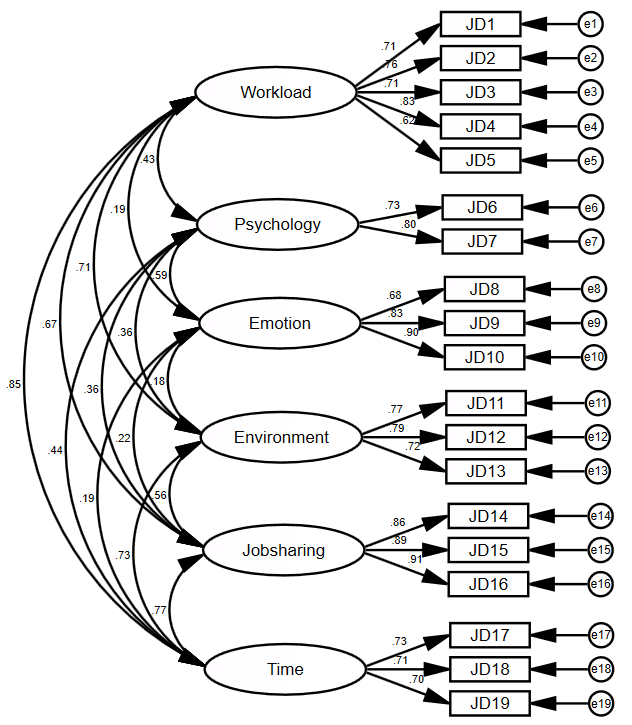


**Figure 1** Confirmatory factor analysis of job demands scale


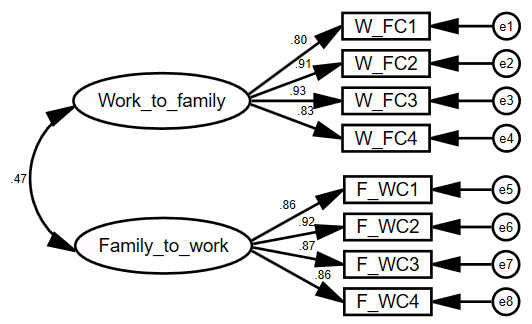


**Figure 2** Confirmatory factor analysis of work-family conflict scale


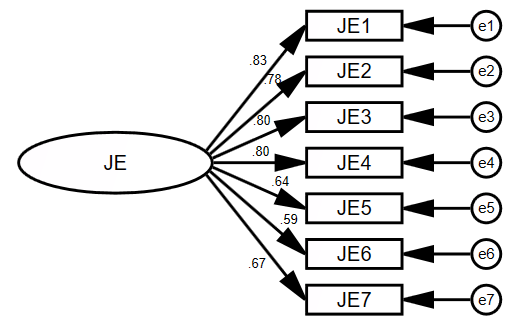


**Figure 3** Confirmatory factor analysis of global job embeddedness scale


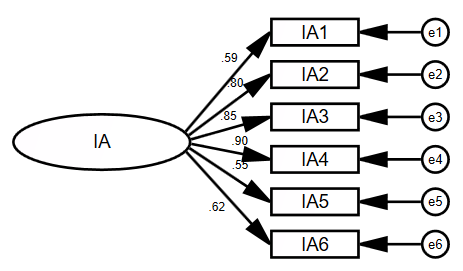


**Figure 4** Confirmatory factor analysis of Stanford presenteeism scale
